# Supplementary material for: Unusual 1-3 peptidoglycan cross-links in Acetobacteraceae are made by L,D-transpeptidases with a catalytic domain distantly related to YkuD domains
Source: J Biol Chem. 2023 Nov 23;300(1):105494. doi: 10.1016/j.jbc.2023.105494 (PMC10727944; doi:10.1016/j.jbc.2023.105494)
Supplement: Supporting Figures S1–S3 [file mmc1.pdf]

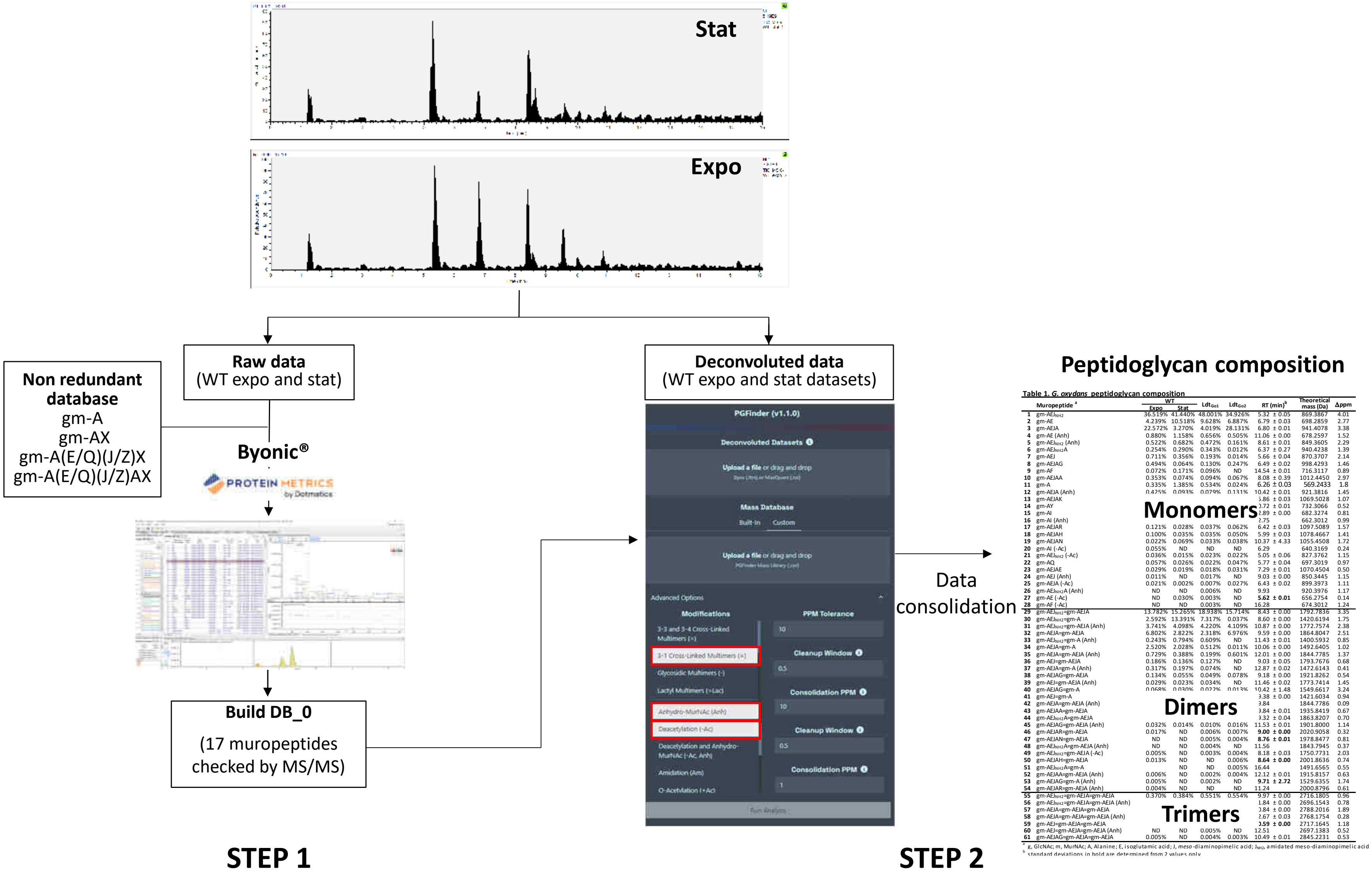

Fig. S1. Strategy for *G. oxydans* peptidoglycan structural analysis. A first search was performed using the Byonic™ module from the Byos® software to identify monomers based on MS/MS data. The search space contained disaccharide substituted by mono-, di-, tri-, tetra- and pentapeptides stems containing glutamic acid (E) or glutamine (Q) in position 2, *meso*-diaminopimelic acid (J) or amidated *meso*-diaminopimelic acid (Z) in position 3, any possible aminoacids (X) in position 4 or the AX dipeptides in positions 4 and 5. The 17 monomers identified by MS/MS were combined to generate the database DB_0. A second search was performed with PGFinder, enabling the formation of dynamic libraries containing dimers and trimers resulting from 4-3, 3-3 and 1-3 cross-links as well as their derivatives containing deacetylated or anhydroMurNAc sugar moieties. The corresponding options are boxes in red in the graphic user interface.

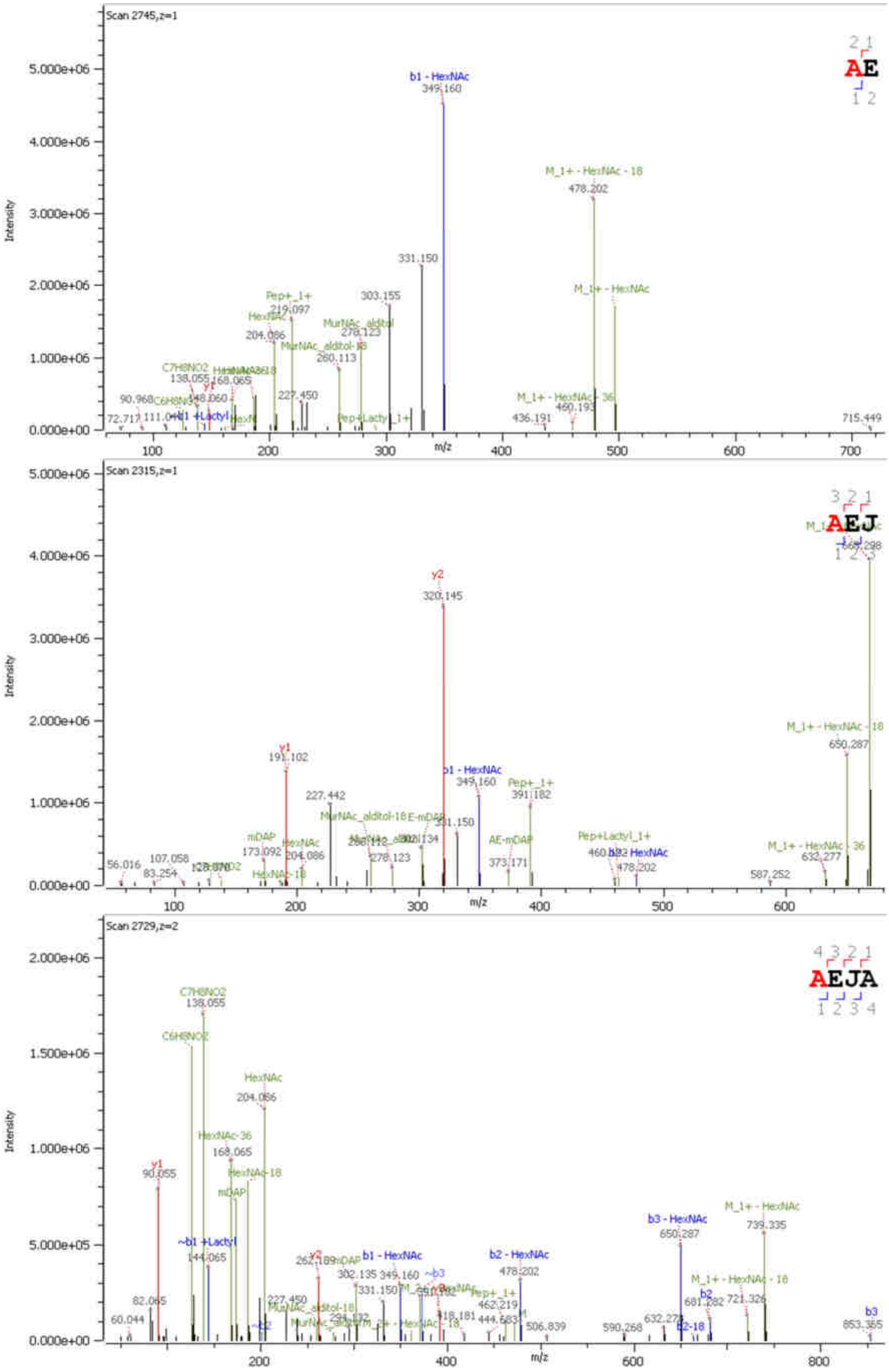
Scan 2745,z=1
Intensity
m/z
AE
b1 - HexNAc
349.160
M_1+ - HexNAc - 18
478.202
331.150
303.155
M_1+ - HexNAc
Pep+_1+
219.097
HexNAc
204.086
MurNAc_alditol
278.123
MurNAc_alditol-18
260.113
C7H8NO2
138.055
168.065
227.450
90.968
148.060
72.717
111.044
M_1+ - HexNAc - 36
460.193
436.191
715.449
Scan 2315,z=1
AEJ
y2
320.145
y1
191.102
227.442
b1 - HexNAc
349.160
Pep+_1+
391.182
M_1+ - HexNAc - 18
650.287
668.298
331.150
AE-mDAP
373.171
mDAP
173.092
HexNAc
204.086
HexNAc-18
278.123
Pep+Lactyl_1+
b2 - HexNAc
478.202
M_1+ - HexNAc - 36
632.277
587.252
56.016
107.058
83.254
Scan 2729,z=2
AEJA
C7H8NO2
138.055
C6H8NO2
HexNAc
204.086
HexNAc-36
168.065
HexNAc-18
mDAP
y1
90.055
~b1 +Lactyl
144.065
b1 - HexNAc
349.160
b2 - HexNAc
478.202
b3 - HexNAc
650.287
M_1+ - HexNAc
739.335
M_1+ - HexNAc - 18
721.326
b2
681.282
b3
853.365
302.135
331.150
Pep+_1+
462.219
418.181
444.683
506.839
590.268
632.27
227.450
MurNAc_alditol-18
82.065
60.044

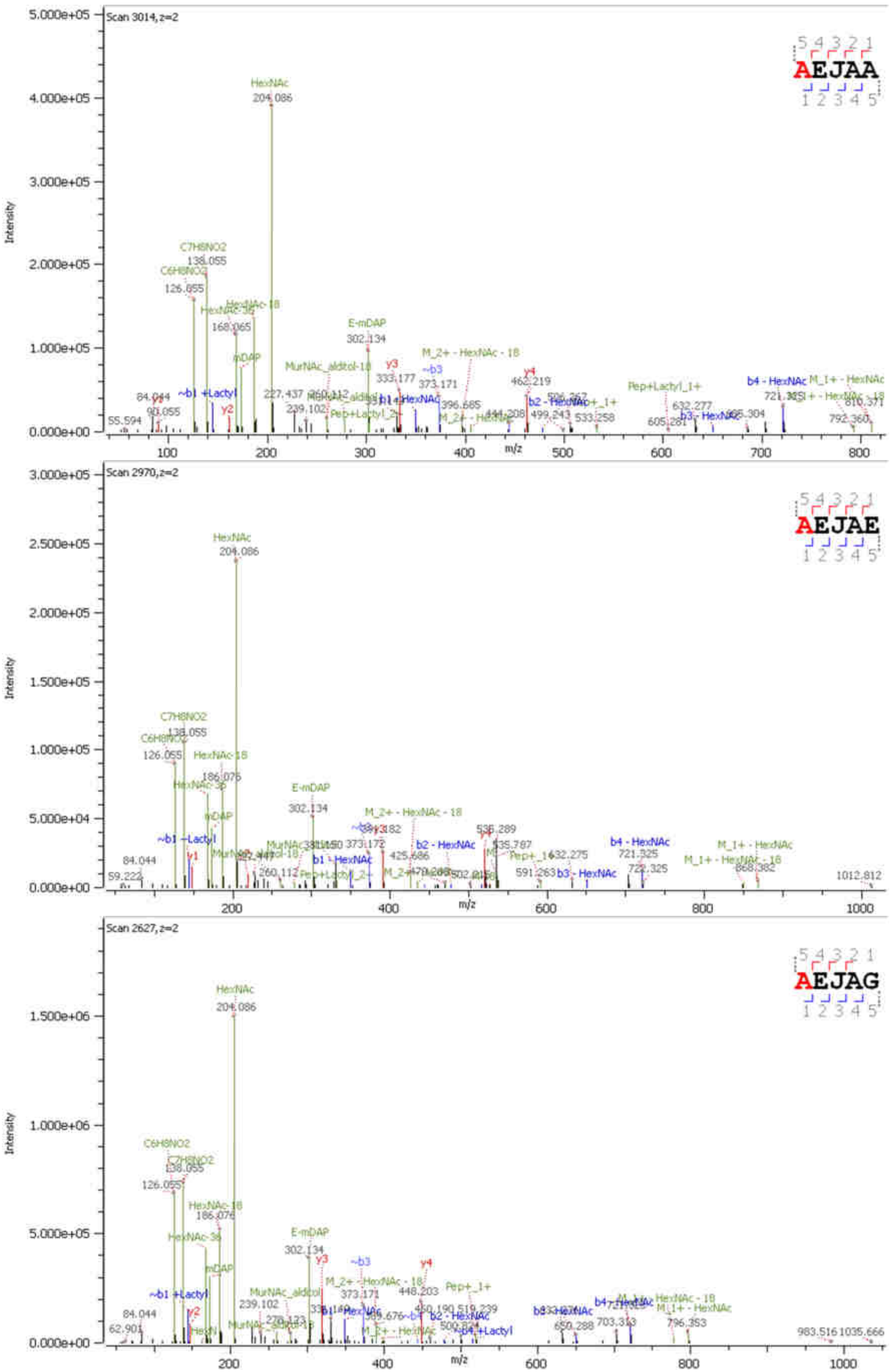
Scan 3014, z=2
AEJAA
Scan 2970, z=2
AEJAE
Scan 2627, z=2
AEJAG
Intensity
m/z
HexNAc
204.086
C7H8NO2
138.055
C6H8NO2
126.055
HexNAc-18
HexNAc-36
mDAP
E-mDAP
302.134
MurNAc_alditol-18
M_2+ - HexNAc - 18
b4 - HexNAc
M_1+ - HexNAc
Pep+Lactyl_1+
b2 - HexNAc
b3 - HexNAc
~b1 +Lactyl

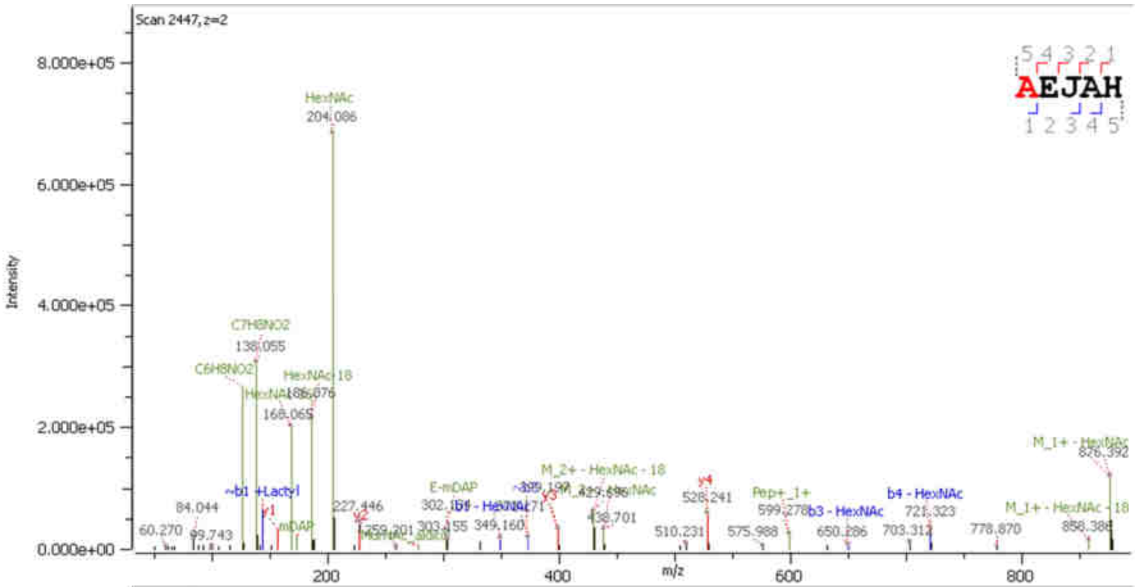
Scan 2447, z=2
AEJAH
5 4 3 2 1
1 2 3 4 5
Intensity
m/z
HexNAc
204.086
C7H8NO2
138.055
C6H8NO2
HexNAc-18
168.065
~b1 +Lactyl
y1
mDAP
227.446
E-mDAP
M_2+ - HexNAc - 18
y4
528.241
Pep+_1+
599.278
b3 - HexNAc
650.286
b4 - HexNAc
721.323
703.312
778.870
M_1+ - HexNAc
876.392
M_1+ - HexNAc - 18
858.386
84.044
60.270
349.160
438.701
510.231
575.988
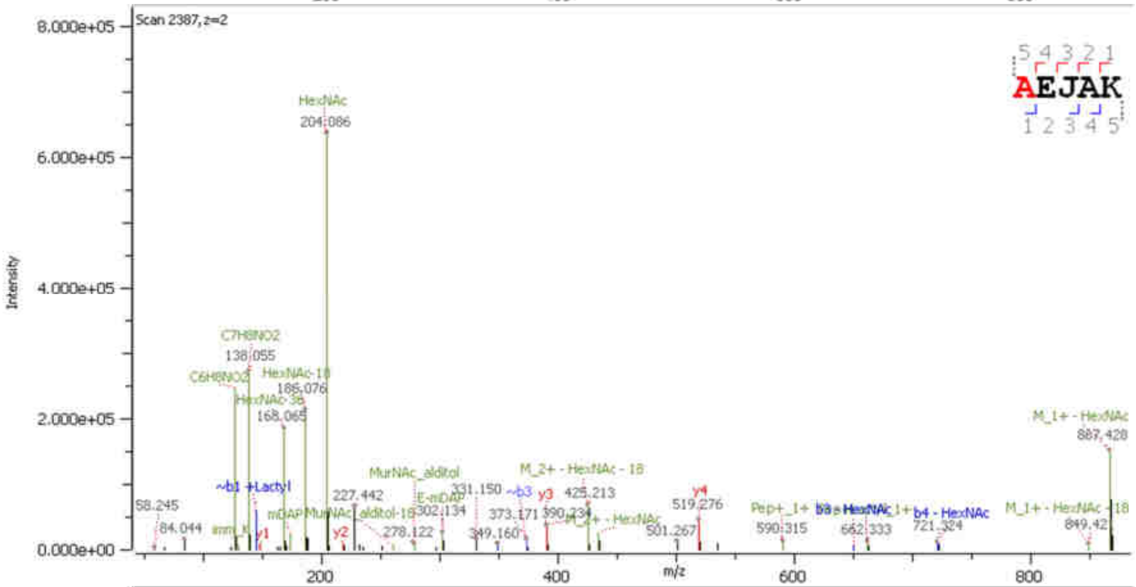
Scan 2387, z=2
AEJAK
5 4 3 2 1
1 2 3 4 5
Intensity
m/z
HexNAc
204.086
C7H8NO2
138.055
C6H8NO2
HexNAc-18
186.076
HexNAc-36
168.065
~b1 +Lactyl
y1
mDAP
MurNAc_alditol
227.442
E-mDAP
302.134
278.122
331.150
~b3
y3
425.213
M_2+ - HexNAc - 18
373.171
390.234
349.160
501.267
y4
519.276
Pep+_1+
590.315
662.333
b4 - HexNAc
721.324
M_1+ - HexNAc
887.428
M_1+ - HexNAc - 18
849.42
58.245
84.044
y2
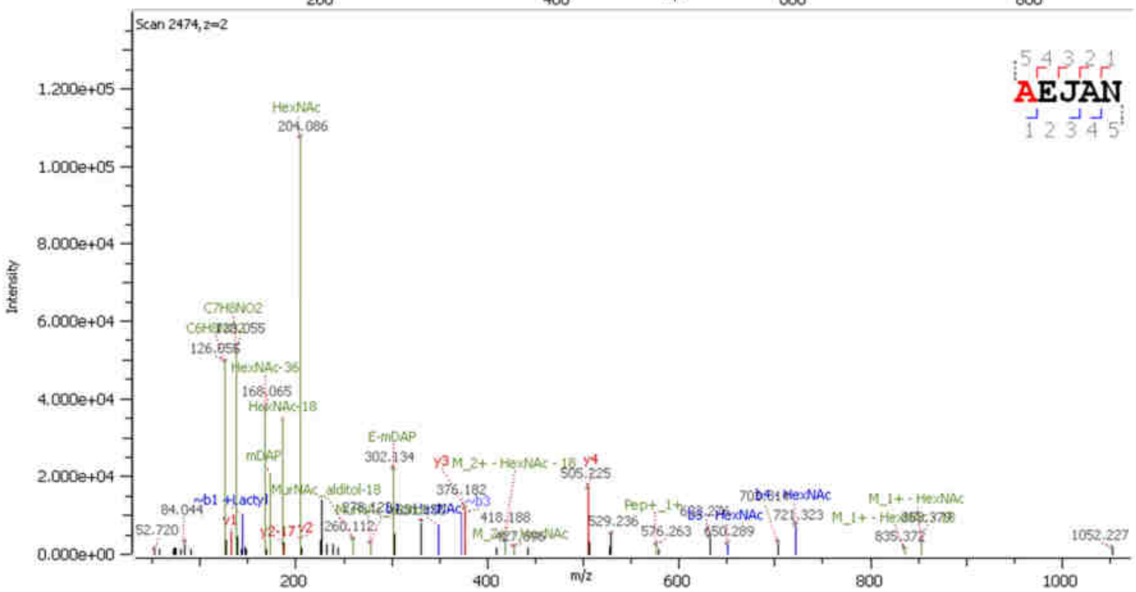
Scan 2474, z=2
AEJAN
5 4 3 2 1
1 2 3 4 5
Intensity
m/z
HexNAc
204.086
C7H8NO2
126.056
HexNAc-36
168.065
HexNAc-18
mDAP
MurNAc_alditol-18
E-mDAP
302.134
y3
M_2+ - HexNAc - 18
y4
505.225
376.182
~b3
418.188
260.112
529.236
Pep+_1+
576.263
650.289
b3 - HexNAc
721.323
M_1+ - HexNAc
835.372
1052.227
52.720
84.044
~b1 +Lactyl
y1
y2-17
y2

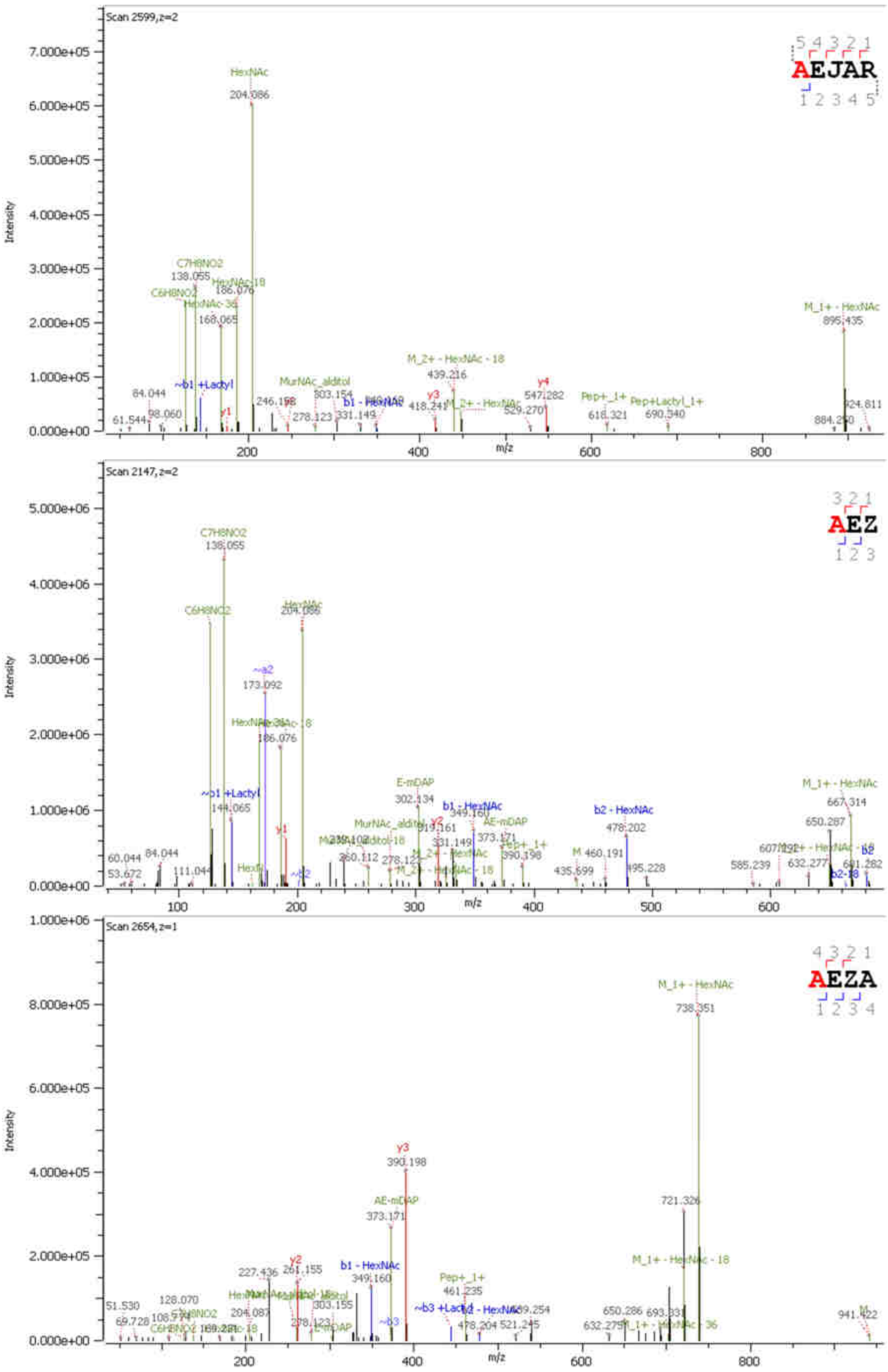
Scan 2599, z=2
AEJAR
HexNAc
204.086
C7H8NO2
138.055
C6H8NO2
186.076
168.065
~b1 +Lactyl
MurNAc_alditol
303.154
M_2+ - HexNAc - 18
439.216
y4
547.282
Pep+_1+
618.321
Pep+Lactyl_1+
690.340
M_1+ - HexNAc
895.435
884.250
924.811
Intensity
m/z
Scan 2147, z=2
AEZ
C7H8NO2
138.055
C6H8NO2
HexNAc
204.086
~a2
173.092
186.076
~b1 +Lactyl
144.065
E-mDAP
302.134
b1 - HexNAc
349.160
AE-mDAP
373.171
b2 - HexNAc
478.202
495.228
M_1+ - HexNAc
667.314
650.287
632.277
585.239
Scan 2654, z=1
AEZA
M_1+ - HexNAc
738.351
y3
390.198
AE-mDAP
373.171
721.326
M_1+ - HexNAc - 18
y2
261.155
b1 - HexNAc
349.160
227.436
Pep+_1+
461.235
303.155
204.087
128.070
51.530
69.728
650.286
941.422

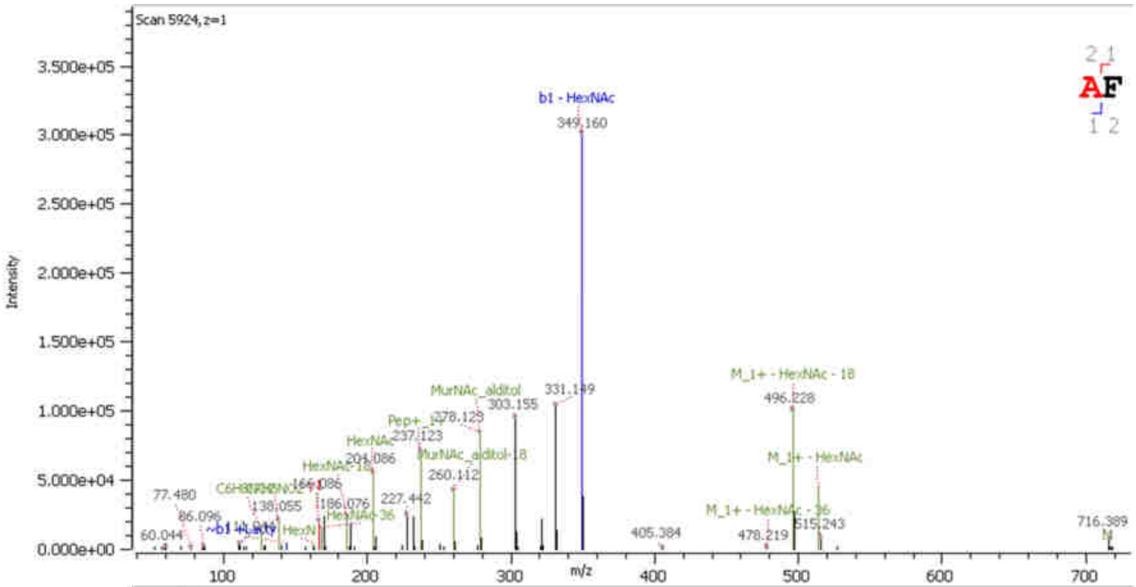
Scan 5924, z=1
Intensity
m/z
b1 - HexNAc
349.160
331.149
303.155
MurNAc_alditol
278.123
Pep+_1+
237.123
HexNAc
204.086
MurNAc_alditol-18
260.112
HexNAc-18
227.442
186.076
HexNAc-36
138.055
77.480
86.096
60.044
M_1+ - HexNAc - 18
496.228
M_1+ - HexNAc
M_1+ - HexNAc - 36
515.243
478.219
405.384
716.389
M
AF

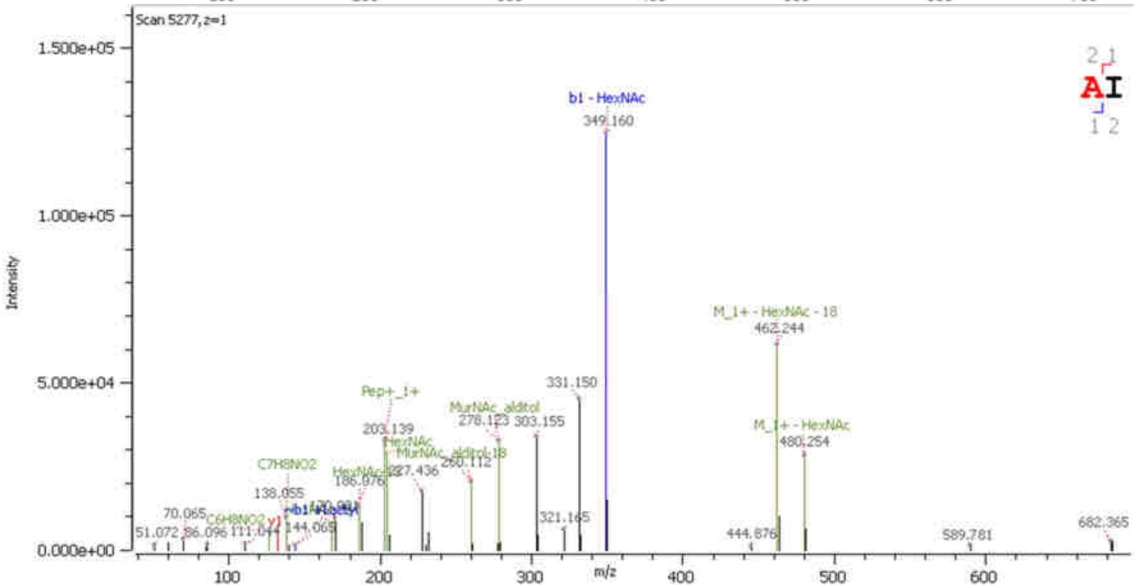
Scan 5277, z=1
Intensity
m/z
b1 - HexNAc
349.160
331.150
303.155
321.165
MurNAc_alditol
278.123
MurNAc_alditol-18
260.112
Pep+_1+
203.139
HexNAc
227.436
186.976
C7H8NO2
138.055
C6H8NO2
70.065
51.072
86.096
111.044
144.065
M_1+ - HexNAc - 18
462.244
M_1+ - HexNAc
480.254
444.876
589.781
682.365
AI

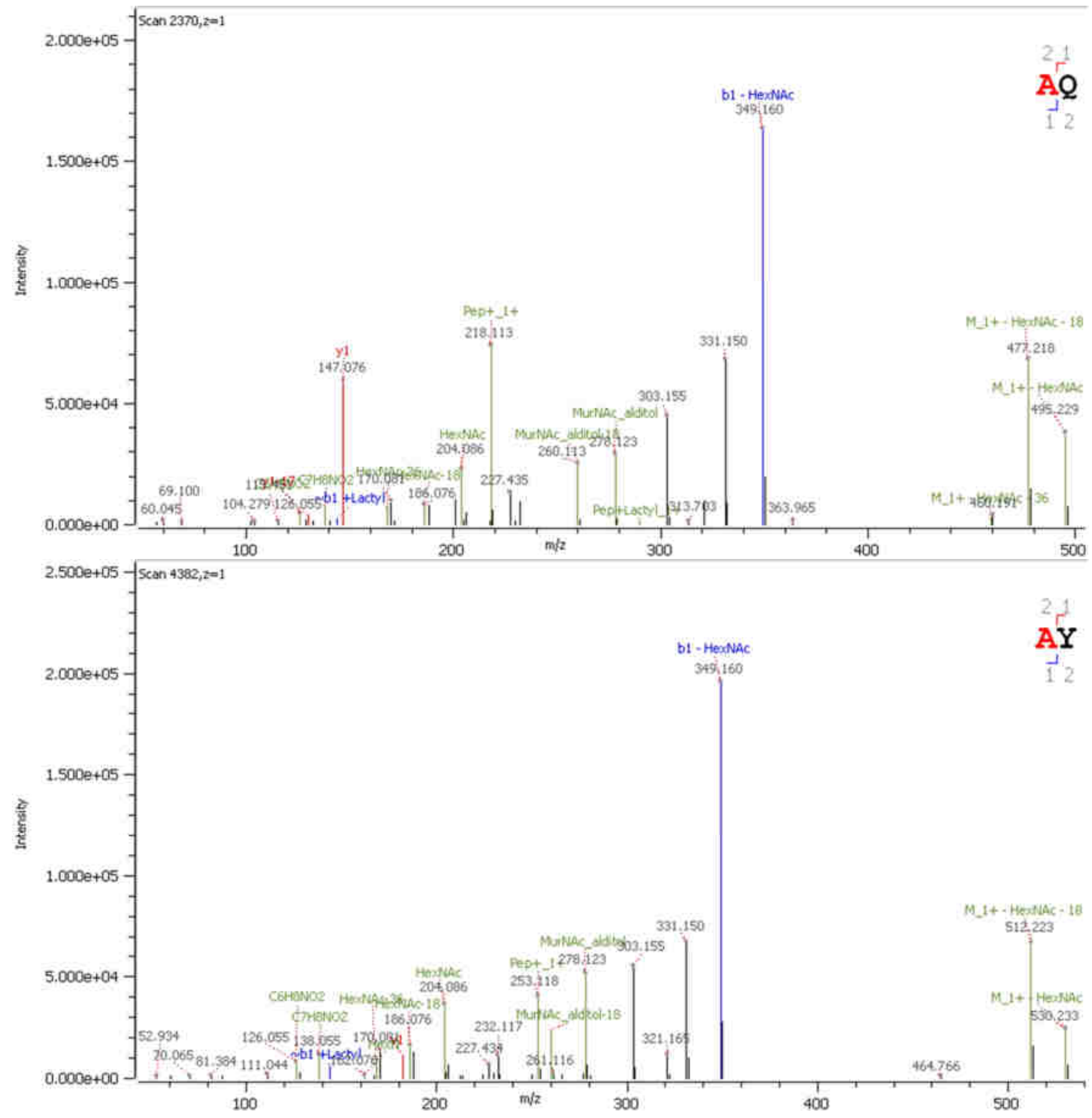

Figure S2. Tandem mass spectrometry analysis of *G. oxydans* monomers using the Byonic™ module.

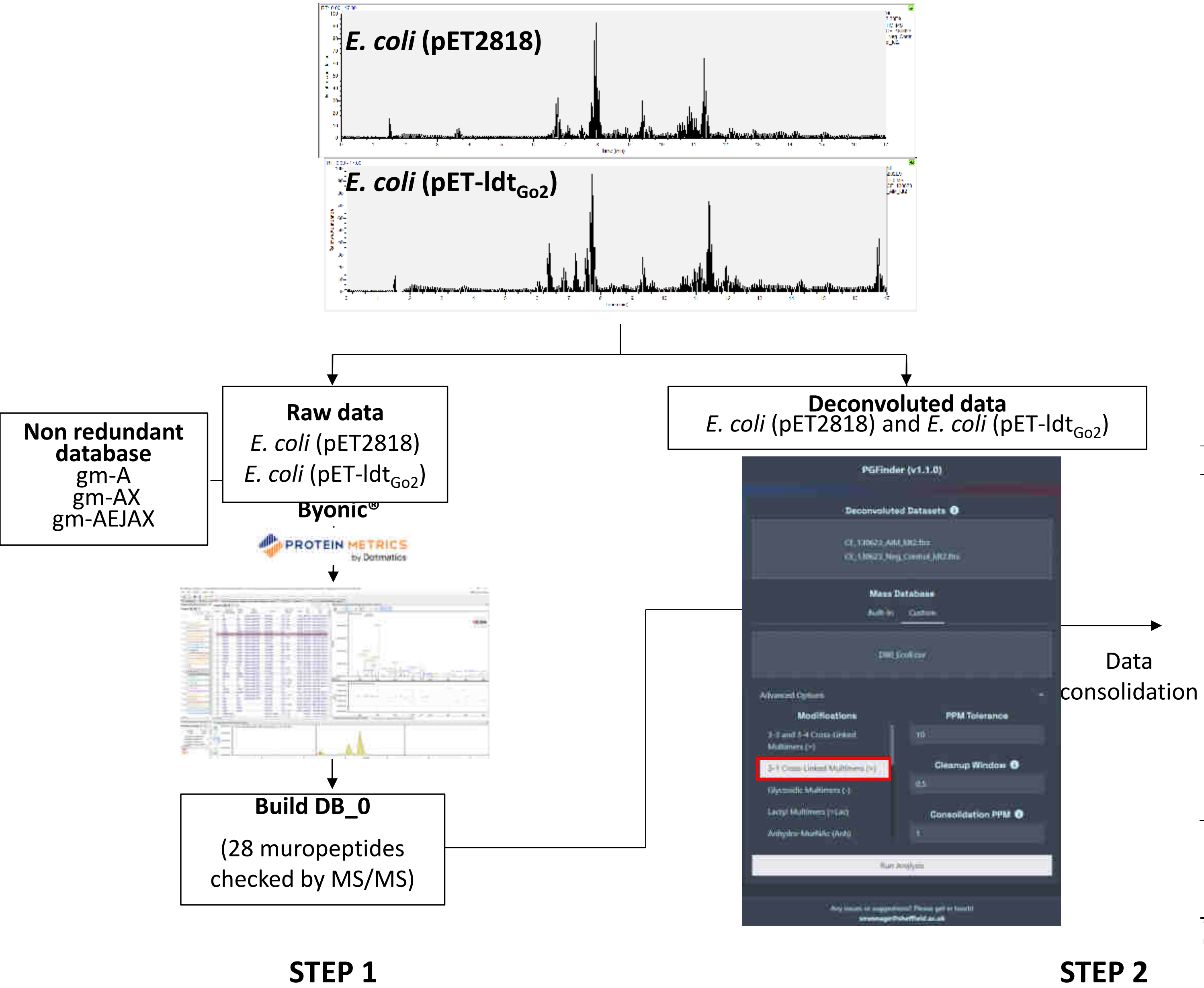

Peptidoglycan composition

	Muropeptides [a]	Strain		RT (min) [b]	Δppm
		E. coli	*E. coli + ldt2*		
1	gm-AEJA	44.981%	28.108%	7.82 ± 0.12	2.4
2	gm-AEJ	17.658%	13.974%	6.57 ± 0.19	3.1
3	gm(-Ac)-AEJA	11.167%	22.256%	7.37 ± 0.14	2.2
4	gm-AEJM	5.850%	0.063%	11.08 ± 0.00	1.0
5	gm-AE	4.220%	2.666%	7.76 ± 0.12	2.1
6	gm-AEJG	3.567%	1.513%	6.91 ± 0.16	1.7
7	gm-AI	ND	0.785%	14.74	1.0
8	gm-AY	ND	0.675%	12.41	0.3
9	gm-AW	ND	0.655%	18.84	0.8
10	gm-AEJN	2.505%	0.235%	6.42 ± 0.21	1.5
11	gm(-Ac)-AEJ	2.289%	5.624%	6.27 ± 0.21	1.9
12	gm-AEJF	2.264%	0.229%	15.10 ± 0.02	1.3
13	gm-AEJK	[illegible]	[illegible]	[illegible]33 ± 0.22	1.4
14	gm-AEJH	[illegible]	[illegible]	[illegible]53 ± 0.23	1.4
15	gm-AEJD	[illegible]	[illegible]	[illegible].93 ± 0.16	1.8
16	gm-A	0.437%	9.038%	6.99 ± 0.15	1.6
17	gm-AEJT	0.364%	0.058%	7.06 ± 0.14	1.1
18	gm-AEJAK	0.341%	0.146%	7.53 ± 0.12	0.6
19	gm-AEJY	0.306%	0.016%	12.40 ± 0.01	0.4
20	gm-AQ	0.273%	0.123%	6.66 ± 0.17	2.0
21	gm-AEJV	0.222%	ND	11.08	0.5
22	gm-AEJAA	0.188%	1.465%	9.07 ± 0.31	1.3
23	gm-AEJI	0.176%	0.008%	14.21 ± 0.65	0.7
24	gm-AEJW	0.173%	0.017%	17.39 ± 0.20	0.6
25	gm-AK	ND	0.052%	5.91	0.9
26	gm-AEJAI	0.051%	0.014%	15.53 ± 0.04	0.4
27	gm-AEJAG	0.035%	0.198%	7.53 ± 0.14	0.7
28	gm-AF	ND	2.177%	16.76	1.7
29	gm-AEJA=gm-A	0.056%	6.437%	11.96 ± 0.07	0.4
30	gm(-Ac)-AEJA=gm-A	ND	2.445%	11.68	1.0
31	gm-AEJ=gm-A	ND	0.422%	11.23	1.3
32	gm-AEJG=gm-A	[illegible]	[illegible]	[illegible].89	0.2
33	gm(-Ac)-AEJ=gm-A	[illegible]	[illegible]	[illegible].89	1.3
34	gm-AEJN=gm-A	ND	[illegible]	10.17	0.5
35	gm-AEJF=gm-A	ND	0.012%	18.39	1.5
36	gm-AEJI=gm-A	ND	0.006%	17.14	1.1

Monomers

Dimers

[a] g, GlcNAc; m, MurNAc; A, Alanine; E, isoglutamic acid; J, *meso*-diaminopimelic acid; J_{NH2}, amidated meso-diaminopimelic acid

[b] standard deviations are determined from 2 values only

Fig. S3. Strategy for peptidoglycan structural analysis of *E. coli* producing Ldt_{Go2}. A first search was performed using the Byonic™ module from Byos® to identify monomers based on MS/MS data. The search space contained disaccharide substituted by mono-, di-, tri-, tetra- and pentapeptides stems containing glutamic acid (E) or glutamine (Q) in position 2, *meso*-diaminopimelic acid (J) or amidated *meso*-diaminopimelic acid (Z) in position 3, any possible aminoacids (X) in position 4 or the AX dipeptides in positions 4 and 5. The 28 monomers identified by MS/MS were combined to generate the database DB_0_Ec. A second search was performed with PGFinder, enabling the formation of dynamic libraries containing dimers and trimers resulting from 1-3 cross-links only (boxed in red).
