# Supplementary material for: Unusual 1-3 peptidoglycan cross-links in Acetobacteraceae are made by L,D-transpeptidases with a catalytic domain distantly related to YkuD domains
Source: J Biol Chem. 2023 Nov 23;300(1):105494. doi: 10.1016/j.jbc.2023.105494 (PMC10727944; doi:10.1016/j.jbc.2023.105494)
Supplement: Supporting Tables S1–S6 [file mmc4.pdf]

Table S1. List of peptide sequences used for *G. oxydans* MS/MS searches

A
AE
AA
AC
AR
AN
AD
AQ
AG
AH
AI
AL
AK
AM
AF
AP
AS
AT
AW
AY
AV
AEJ
AEJA
AEJC
AEJD
AEJE
AEJF
AEJG
AEJH
AEJI
AEJK
AEJM
AEJN
AEJP
AEJQ
AEJR
AEJS
AEJT
AEJV
AEJW
AEJY
AEJAA
AEJAC
AEJAD
AEJAE
AEJAF
AEJAG
AEJAH
AEJAI
AEJAK
AEJAM
AEJAN
AEJAP
AEJAQ
AEJAR
AEJAS
AEJAT
AEJAV
AEJAW
AEJAY
AEZ
AEZA
AEZC
AEZD
AEZE
AEZF
AEZG
AEZH
AEZI
AEZK
AEZM
AEZN
AEZP
AEZQ
AEZR
AEZS
AEZT
AEZV
AEZW
AEZY
AEZAA
AEZAC
AEZAD
AEZAE
AEZAF
AEZAG
AEZAH
AEZAI
AEZAK
AEZAM
AEZAN
AEZAP
AEZAQ
AEZAR
AEZAS
AEZAT
AEZAV
AEZAW
AEZAY
AQJ
AQJA
AQJC
AQJD
AQJE
AQJF
AQJG
AQJH
AQJI
AQJK
AQJM
AQJN
AQJP
AQJQ
AQJR
AQJS
AQJT
AQJV
AQJW
AQJY
AQJAA
AQJAC
AQJAD
AQJAE
AQJAF
AQJAG
AQJAH
AQJAI
AQJAK
AQJAM
AQJAN
AQJAP
AQJAQ
AQJAR
AQJAS
AQJAT
AQJAV
AQJAW
AQJAY
AQZ
AQZA
AQZC
AQZD
AQZE
AQZF
AQZG
AQZH
AQZI
AQZK
AQZM
AQZN
AQZP
AQZQ
AQZR
AQZS
AQZT
AQZV
AQZW
AQZY
AQZAA
AQZAC
AQZAD
AQZAQ
AQZAF
AQZAG
AQZAH
AQZAI
AQZAK
AQZAM
AQZAN
AQZAP
AQZAQ
AQZAR
AQZAS
AQZAT
AQZAV
AQZAW
AQZAY

Table S2. Database for *G. oxydans* PGFinder searches

Structure	Monoisotopicmass
gm-A|1	569.24331
gm-AE|1	698.2859
gm-AF|1	716.31172
gm-AI|1	682.32737
gm-AQ|1	697.30189
gm-AY|1	732.30664
gm-AEJ|1	870.37069
gm-AEZ|1	869.38668
gm-AEJA|1	941.40783
gm-AEZA|1	940.42382
gm-AEJAA|1	1012.44497
gm-AEJAE|1	1070.45042
gm-AEJAG|1	998.42929
gm-AEJAH|1	1078.46674
gm-AEJAK|1	1069.50279
gm-AEJAN|1	1055.45076
gm-AEJAR|1	1097.50894

Table S3. List of peptide sequences used for *E. coli* MS/MS searches

A
AE
AC
AD
AF
AG
AH
AI
AJ
AK
AL
AM
AN
AP
AQ
AR
AS
AT
AV
AW
AY
AZ
AEJ
AEJA
AEJC
AEJD
AEJE
AEJF
AEJG
AEJH
AEJI
AEJK
AEJM
AEJN
AEJP
AEJQ
AEJR
AEJS
AEJT
AEJV
AEJW
AEJY
AEJAA
AEJAC
AEJAD
AEJAE
AEJAF
AEJAG
AEJAH
AEJAI
AEJAK
AEJAM
AEJAN
AEJAP
AEJAQ
AEJAR
AEJAS
AEJAT
AEJAV
AEJAW
AEJAY

Table S4. Database for *E. coli* PGFinder searches

Structure	Monoisotopic mass
gm-AE|1	698.2858
gm-AF|1	716.3116
gm-AI|1	682.3273
gm-AK|1	697.3382
gm-AQ|1	697.3018
gm-AW|1	755.3225
gm-AY|1	732.3065
gm-AEJ|1	870.3706
gm-AEJA|1	941.4077
gm-AEJD|1	985.3975
gm-AEJF|1	1017.439
gm-AEJG|1	927.392
gm-AEJH|1	1007.4295
gm-AEJI|1	983.4546
gm-AEJK|1	998.4655
gm-AEJM|1	1001.4111
gm-AEJN|1	984.4135
gm-AEJT|1	971.4183
gm-AEJV|1	969.439
gm-AEJW|1	1056.4499
gm-AEJY|1	1033.4339
gm-AEJAA|1	1012.4448
gm-AEJAG|1	998.4292
gm-AEJAI|1	1054.4918
gm-AEJAK|1	1069.5027
gm(-Ac)-AEJ|1	828.36008
gm(-Ac)-AEJA|1	899.39722
gm-A|1	569.24331

Table S5. Species, taxids, and genome accessions used for comparative genomics.

Species	Taxid	Accession
Escherichia coli	562	GCF_000005845.2
Clostridioides difficile	1496	GCF_018885085.1
Mycobacterium tuberculosis	1773	GCF_000195955.2
Enterococcus faecium	1352	GCF_009734005.1
Sphingomonas paucimobilis	13689	GCF_016027095.1
Erythrobacter litoralis	39960	GCF_001719165.1
Caulobacter vibrioides	155892	GCF_000022005.1
Asticcacaulis biprosthecum	76891	GCF_000204015.1
Roseobacter denitrificans	2434	GCF_002983865.1
Rhodobacter sphaeroides	1063	GCF_000021005.1
Bartonella grahamii	33045	GCF_000022725.1
Labrys okinawaensis	346911	GCF_002982075.1
Aquamicrobium aerolatum	561088	GCF_900113935.1
Mesorhizobium mediterraneum	43617	GCF_002284565.1
Agrobacterium tumefaciens	358	GCF_003667905.1
Sinorhizobium meliloti	110321	GCF_007827695.1
Angulomicrobium tetraedale	217068	GCF_014195655.1
Hyphomicrobium denitrificans	53399	GCF_000143145.1
Hirschia baltica	2724	GCF_000023785.1
Hyphomonas sediminis	2866160	GCF_019679475.1
Magnetospirillum gryphiswaldense	55518	GCF_000513295.1
Thalassospira lucentensis	168935	GCF_000421265.1
Roseomonas gilardii	257708	GCF_001941945.1
Acidiphilium facilis	525	GCF_000687875.1
Acidomonas methanolica	437	GCF_004346035.1
Komagataeibacter xylinus	28448	GCF_004006375.1
Gluconobacter oxydans	442	GCF_000583855.1
Gluconobacter frateurii	38308	GCF_002723955.1
Acetobacter pasteurianus	438	GCF_009914215.2
Acetobacter tropicalis	104102	GCF_001580945.1
Acetobacter pomorum	65959	GCF_002738225.1
Acetobacter aceti	435	GCF_000379545.1

Table S6. Bacterial strains, plasmids, and oligonucleotides.

Strains/plasmids/ oligonucleotides	Relevant properties/sequence [a]	Source
**Strains**		
Gluconobacter oxydans		
B58	*G. oxydans* reference strain (ATCC NRLL B58)	ATCC
621H	*G. oxydans* reference strain (ATCC 621H)	ATCC
B58 ldt_{Go1}	B58 with a transposon insertion in Go2094 (GOX2269 in strain 621H); KanR	(1)
B58 ldt_{Go2}	B58 with a transposon insertion in Go2227 (GOX1074 in strain 621H); KanR	(1)
B58 ldt_{Go2} (pBBR-TetR-Go2227)	B58 ldt_{Go2} mutant complemented; KanR GmR	This work
Escherichia coli		
BL21(DE3) Lemo	Expression strain	NEB
BL21(DE3) (pET-Go2227)	BL21(DE3) Lemo expressing Ldt_{Go2} ; AmpR	This work
**Plasmids**		
pET2818	pET28a derivative for recombinant protein expression; AmpR	(2)
pET-Ldt_{Go2}	pET2818 derivative encoding full length Ldt_{Go2}; AmpR	This work
pBBR1MCS-5	pBBR1-MCS derivative; GmR	(3)
pBBR1MCS-5-T_{gdhM}-*tetR-mNG*	pBBR1-MCS5 derivative for inducible expression with anhydrotetracycline; GmR	(4)
pBBR-TetR-Go2227	pBBR1-TetR derivative expressing full length Ldt_{Go2}; GmR	This work
**Oligonucleotides**		
SM_0725	ggctacggtctcccgaagtctcgggccgtctcttgggctt	
SM_0726	ggctacggtctcttccttggattcacttttctctatcactgataggg	
SM_0727	ggctacggtctctaggagatatcatatgcgtgatgtttccagactgac	
SM_0728	ggctacggtctcaaaggtaacggtcttttatccgcaatag	
SM_0729	ggctacggtctcaccttaacgcaaaaaaccccgcttcggcgg	
SM_0730	ggctacggtctctttcgggagcgcctgaagcccgtt	

[a] KanR, resistant to kanamycin; GmR, resistant to gentamicin; AmpR, resistant to ampicillin

(1) Schmitz, A. M., Pian, B. Medin, S., Reid, M. C., Wu, M., Gazel, E., Barstow, B. (2021) Generation of a *Gluconobacter oxydans* knockout collection for improved extraction of rare earth elements. Nat Commun **12**(1): 6693

(2) Eckert, C., Lecerf, M., Dubost, L., Arthur, M. and Mesnage, S. (2006) Functional analysis of AtlA, the major *N*-acetylglucosaminidase of *Enterococcus faecalis*. *J Bacteriol*. **188**(24):8513-8519

(3) Kovach, M. E., Elzer, P. H., Hill, D. S., Robertson, G. T., Farris, M. A., Roop, R. M., Peterson, K. M. (1995) Four new derivatives of the broad-host-range cloning vector pBBR1MCS, carrying different antibiotic-resistance cassettes. *Gene*. **166**(1):175–6.

(4) Fricke, P. M., Lürkens, M., Hünnefeld, M., Sonntag, C. K., Bott, M., Davari, M. D., Polen, T. (2021) Highly tunable TetR-dependent target gene expression in the acetic acid bacterium *Gluconobacter oxydans*. *Appl Microbiol Biotechnol*. **105**(18):6835-6852.
